# Hexokinase2-engineered T cells display increased anti-tumor function

**DOI:** 10.3389/fimmu.2025.1477929

**Published:** 2025-03-20

**Authors:** Raphaëlle Toledano Zur, Shiran Didi Zurinam, Maria Radman, Elia Funaro Balouka, Tatiana Borodianskiy-Shteinberg, Dieter Saur, Cyrille J. Cohen

**Affiliations:** ^1^ The Laboratory of Tumor Immunology and Immunotherapy, The Mina and Everard Goodman Faculty of Life Sciences, Bar-Ilan University, Ramat Gan, Israel; ^2^ Division of Translational Cancer Research, German Cancer Research Center and German Cancer Consortium, Heidelberg and Center for Translational Cancer Research (TranslaTUM), Institute of Experimental Cancer Therapy, Klinikum Rechts der Isar, School of Medicine, Technische Universität München, Munich, Germany

**Keywords:** T-cells, cellular immunotherapy, immunometabolism, hexokinase 2, TCR

## Abstract

**Background:**

T cells face significant metabolic challenges in the tumor microenvironment (TME), where cancer cells monopolize critical nutrients like glucose and amino acids. This metabolic competition supports tumor growth while impairing T-cell anti-tumor responses, partly by reducing glycolytic function. Hexokinase 2 (HK2), a key enzyme in glycolysis, plays a pivotal role in maintaining T-cell functionality.

**Methods:**

To enhance T-cell function, primary human T cells were genetically engineered to overexpress HK2 alongside a tumor-specific receptor. These engineered T cells were tested *in vitro* and *in vivo* to evaluate their metabolic and therapeutic efficacy.

**Results:**

HK2-engineered T cells exhibited increased glycolytic capacity, leading to enhanced cytokine secretion, activation marker expression, and metabolic activity compared to controls. *In vivo* studies using a human tumor xenograft model demonstrated the superior therapeutic efficacy of HK2-engineered T cells, including delayed tumor growth and improved survival.

**Conclusion:**

HK2 overexpression improves T-cell metabolic fitness and functionality in hostile TMEs, offering a promising foundation for the development of next-generation immunotherapies targeting T-cell metabolism.

## 1 Introduction

Over the past decade, various immunotherapeutic strategies have emerged to bolster anti-tumor immunity, prominently including the utilization of immune checkpoint inhibitors (1) and adoptive cell transfer of tumor-reactive T cells (2, 3). Several adoptive cell therapies demonstrated encouraging results in the clinic and encompass the expansion of tumor-infiltrating lymphocytes (TILs) or the genetic modification of peripheral T cells to endow them with tumor-specificity and functional enhancements (4, 5). The latter can be achieved by expressing TCRs or CARs specific for defined tumor antigens, showing promise in different types of cancers (6, 7).

CD8^+^ T cells have been established as key players in controlling tumor progression (8) in both human and mice models. However, effective anti-tumor immune responses can be hindered by mechanisms such as tumor-induced immunosuppression and metabolic dysfunction, which manifest through conditions like acidification, hypoxia, the accumulation of immunosuppressive metabolites within the tumor microenvironment (TME) and glucose deprivation (9, 10). The tumor microenvironment (TME) poses significant challenges to T-cell functionality. Tumor cells monopolize critical nutrients like glucose, leading to metabolic competition that hinders T-cell proliferation and cytokine production. Additionally, immune checkpoint pathways, such as PD-1/PD-L1 and CTLA-4, further suppress T-cell activity. Environmental factors like hypoxia, acidic pH, and the accumulation of immunosuppressive metabolites exacerbate these challenges. Consequently, T cells often encounter limitations in accessing essential nutrients crucial for their proliferation and effector functions when engaging with tumor cells (11, 12). Notably, tumors undergo metabolic alterations, such as the “Warburg effect”, which facilitates their growth by enhancing nutrient uptake and metabolic pathway rewiring (13).

Glucose serves as a pivotal nutrient metabolized by all cells to generate energy during periods of rapid growth. In glycolysis, glucose undergoes a 10-step enzymatic conversion process to yield two pyruvate molecules, which are either utilized for aerobic respiration and oxidative phosphorylation (OXPHOS) or alternatively, converted to lactate during aerobic glycolysis (14). Enhanced glucose metabolism following T cell activation is paramount for triggering rapid proliferation through metabolite synthesis (11). Additionally, glycolysis in T cells plays a crucial role in producing key effector molecules such as IFNγ, with glycolytic metabolites like phosphoenolpyruvate (PEP) potentially acting as checkpoints to facilitate T cell activation (15).

Upon activation, T cells upregulate the expression of glucose transporters and glycolytic enzymes while maintaining aerobic respiration to generate ATP necessary for their functions and proliferation. HK2 is a key glycolysis rate-limiting enzyme that catalyzes the first step of glucose metabolism, phosphorylating glucose to glucose 6-phosphate (G6P) (16). HK2 acts as a bottleneck enzyme involved in cell proliferation, regulating intracellular mitochondria Ca2^+^ fluxes, autophagy, and inhibiting cell death independently (17).

As reduced glycolysis may impair T-cell function in the tumor microenvironment, we hypothesize that it may be possible to counter this by promoting glucose metabolism via the increased expression of HK2. Specifically, we genetically enforced the expression of hexokinase 2 (HK2) in melanoma specific T-cells which led to an augmented glycolytic, oxygen consumption rates, and ATP production in T cells. Ultimately, our findings demonstrate that the expression of HK2 significantly enhances anti-tumor function both *in vitro* and, importantly, in a xenograft model of human tumors.

## 2 Materials and methods

### 2.1 Patient PBMCs and cell lines

Peripheral blood mononuclear cells (PBMCs) were obtained from healthy donors from the Israeli Blood Bank, after the donors signed an informed consent form. The studies were conducted in accordance with the local legislation and institutional requirements. Melanoma cell lines HLA-A2^+^/MART-1^+^ (624.38) and HLA-A2^−^/MART-1^+^ (888) were generated at the Surgery Branch (NCI, NIH, MD), as described previously (18). 888A2 is an HLA-A*0201-transduced line derived from 888. SK-MEL23 is an HLA-A2^+^ melanoma cell line (CVCL_6027). A375 (CVCL_0132) melanoma is HLA-A2^+^/MART-1^-^. Adherent cells were cultured in DMEM (Sartorius, Germany), supplemented with 10% heat-inactivated Fetal Bovine Serum (Biological Industries, Beth Haemek, Israel). Human lymphocytes were cultured in a mix of RPMI 1640 (Merck, Germany) and TexMacs media (Miltenyi Biotech), supplemented with 10% heat-inactivated Fetal Bovine Serum, 1% L-Glutamine, 1% Pen-Strep solution, 0.01M HEPES, and 300 IU/ml IL-2 (Peprotech, Israel). All cells were maintained at 37°C and 5% CO_2_.

### 2.2 Transduction of PBLs and retroviral constructs

Retroviral transduction was performed as previously described using Retronectin (Takara, Japan) (7, 18). The α and β chains from the MART-1-specific F4 TCR were subcloned into the MSGV1 vector (18). As a control, we used a truncated version of NGFR (18), a widely used inert marker in T-cell engineering due to its lack of signaling domains and non-interference with cellular function used for efficient tracking and selection of transduced cells (19–22).The cDNA encoding HK2 was purchased (SinoBiological) and subcloned into the MSGV1 vector. PBLs were stimulated in the presence of 50ng/ml OKT3 (eBioscience, San Diego, CA). 3 days after stimulation, lymphocytes were transduced with the F4 TCR, and after 24 h, with viral supernatant encoding HK2 or control.

### 2.3 Gene-expression analysis by real-time PCR

The RNA was extracted according to the manufacturer’s instructions of the Total RNA Mini Kit from Geneaid. cDNA was synthesized using the iScript cDNA Synthesis kit (Biorad). qPCR was performed with SYBR Green ER qPCR (Hylabs, Israel). RNA levels were normalized to b-actin. Primer sequences that were used for real-time PCR are as follow: **b-actin** For: 5’CTGTACGCCAACACAGTGCT; Rev: 5’GCTCAGGAGGAGCAATGATC and **HK2** For: 5’CAAGAAGCTCCCACTGGGTT; Rev:5’CAACGTCTCTGCCTTCCACT.

### 2.4 Antibodies and flow cytometry

Fluorophore-labeled anti-human NGFR, CD8, CD4, CD3, CD137, TIM3, LAG3, CD69, CD25 CCR7, and CD45RO were purchased from BioLegend (San Diego, CA, USA). Anti-Vβ12 antibody (Beckman Coulter Cat# IM2291) is specific for F4 TCR. Cells were stained in a FACS buffer made of PBS, 0.5% BSA, and 0.02% sodium azide for 30 min. For intracellular staining, we used an HK2-specific antibody (ab209847). Proliferation assays using CFSE were performed as previously described (18). Cells were analyzed by flow cytometry, gated on the live population as described.

### 2.5 Cytokine release and cytotoxicity assays

Cytokine release assays were measured using commercially available human ELISA kits for IL-2, IFNγ, TNFα and Granzyme (R&D Systems, Minneapolis, MN, USA). 1x10^5^ responder cells (T cells) and 1x10^5^ target cells were incubated for 16 hours in a 200 μL culture volume in individual wells of 96-well plates. For cytotoxicity, 1x10^4^ mCherry expressing target cells were seeded on a flat 96 plate well and co-cultured with T cells at a ratio of 1:1 to 1:10 E:T for 24h in an Incucyte apparatus and analyzed for orange integrated intensity.

### 2.6 Metabolism-related assays

ATP levels were measured using CellTiter-Glo luminescent cell viability assay (Promega), according to the manufacturer’s instructions. Mitochondrial staining was performed using Mitospy Green FM (BioLegend). Extracellular acidification rate was measured from cells in non-buffered DMEM containing 5mM glucose, 2mM L-glutamine, and 1mM sodium pyruvate, under basal conditions in response to glycolysis inhibitors (glucose 11.1 mM, 1.15 uM oligomycin, and 100 mM 2-DG - Sigma-Aldrich) on the SeaHorse XFe96 Extracellular Flux Analyzer (Agilent Technologies). Glucose-6-Phosphate was measured using the Glucose-6-phosphate kit (Abcam, #ab83426) following the manufacturer’s instructions. Oxygen consumption rate (OCR) was measured using the Extracellular Oxygen Consumption Assay (Abcam, #ab197243) following the manufacturer’s instructions.

### 2.7 *In vivo* assay

NSG mice were inoculated with 2 million 888/A2 tumor cells in 100ul HBSS and 100µl Cultrex matrix (Trevigen) in dorsal flank of 6-12-week NSG mice. Upon tumor establishment, mice were randomized and injected in the tail vein with 2 injections of 5x10^6^ transduced lymphocytes on days 7 and 14 after tumor inoculation. Tumor growth was measured every 2-3 days in a blinded fashion using a caliper and calculated using: (Dxd^2^)xπ/6, where D is the largest tumor diameter and d is its perpendicular diameter. All the procedures were approved by the university committee for animal welfare.

### 2.8 Statistical analysis

A paired *Student’s* log t-test was used to determine statistical significance. Data are reported as mean ± SEM. Statistical values, including the number of replicates (n), can be found in the Figure legends. ∗p < 0.05, ∗∗p<0.01, ∗∗∗p < 0.001. Survival curves were compared using a LogRank analysis. The statistical test used for each figure is described in the corresponding legend.

## 3 Results

### 3.1 Human T cells can be engineered to express high levels of HK2

**Figure 1:**
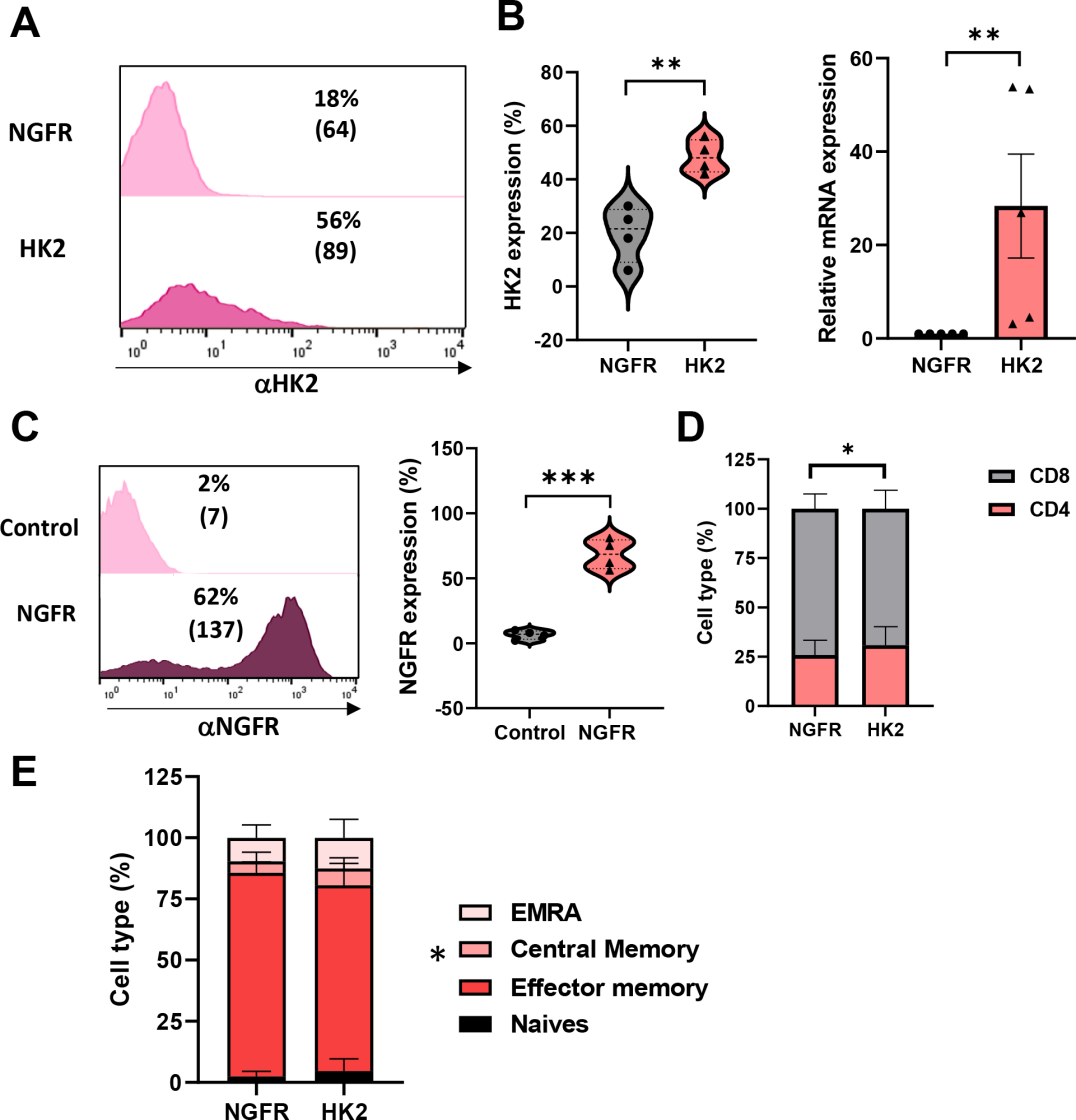
Human T cells can be engineered to express high levels of HK2. **(A–C)** Human PBLs were transduced with a retroviral vector encoding HK2 or NGFR. 72 h after transduction, transgene expression was measured by flow cytometry using antibodies specific for HK2 or NGFR. The left panel is a representative result, and the right panel presents the mean+SEM of n=4 **(C)** RNA from HK2 transduced T cells was extracted and reverse-transcribed. HK2 transcript expression was measured by qPCR and normalized to control. These results are presented as mean+SEM of n=5. **(D)** The CD4/CD8 ratio of transduced cells was determined by flow cytometry. These results are representative of n=8 independent experiments. **(E)** The memory phenotype of transduced cells was determined by flow cytometry based on CD45RO and CCR7 expression. EM - Effector memory (CD45RO+/CCR7-), CM - central memory (CD45RO+/CCR7+), EMRA - terminally differentiated effector memory cells re-expressing CD45RA (CD45RO-/CCR7-) or naïve cell population (CD45RO+/CCR7+). These results are presented as the mean+SEM of n=6. ∗: p < 0.05, ∗∗: p<0.01, ∗∗∗: p < 0.001.

HK2 is a key enzyme involved in cellular metabolism, particularly in the process of glycolysis. It catalyzes the first step of the latter, which involves the phosphorylation of glucose to form glucose-6-phosphate (G6P). Here, primary human T cells were transduced with a retroviral vector encoding for HK2 or truncated NGFR genes (control). We observed a significant increase in HK2 expression with 56% and an MFI (mean fluorescence intensity) of 89 positive cells compared to 18% and an MFI of 64 in the control T cells (basal levels) (**Figure 1A**). This was confirmed using qPCR to evaluate the levels of HK2 transcript in engineered T-cells and we observed close to 30-fold increase in HK2 mRNA (**Figure 1B**). In parallel, we also observed a high transduction level with the control gene (truncated NGFR) compared to untransduced T cells (62% vs. 2% respectively - **Figure 1C**).

Then, we phenotypically characterized the HK2-transduced population. We measured the distribution of CD4^+^/CD8^+^ cells following transduction. We observed a slight increase in CD4^+^ T-cells in the HK2 population compared to control (31% vs. 26% respectively - **Figure 1D**; p=0.025), possibly influenced by differences in the metabolic programming of CD4^+^ and CD8^+^ T cells, since CD4^+^ T cells have been reported to exhibit higher basal glycolytic potential and mitochondrial content compared to CD8^+^ T cells under quiescent conditions (23). Additionally, we assessed the memory phenotype of transduced cells by staining them for CD45RO and CCR7 and dividing them into effector memory, central memory, EMRA (terminally differentiated effector memory cells re-expressing CD45RA), or naïve cell population. We noted a significant difference in the memory phenotype between HK2 and NGFR-transduced T cells with an increase in central memory T cells in the HK2 group (6.88% compared to 4.67% in the control group; p=0.026; **Figure 1E**).

### 3.2 HK2-engineered T cells display increased glycolysis and metabolic fitness

**Figure 2:**
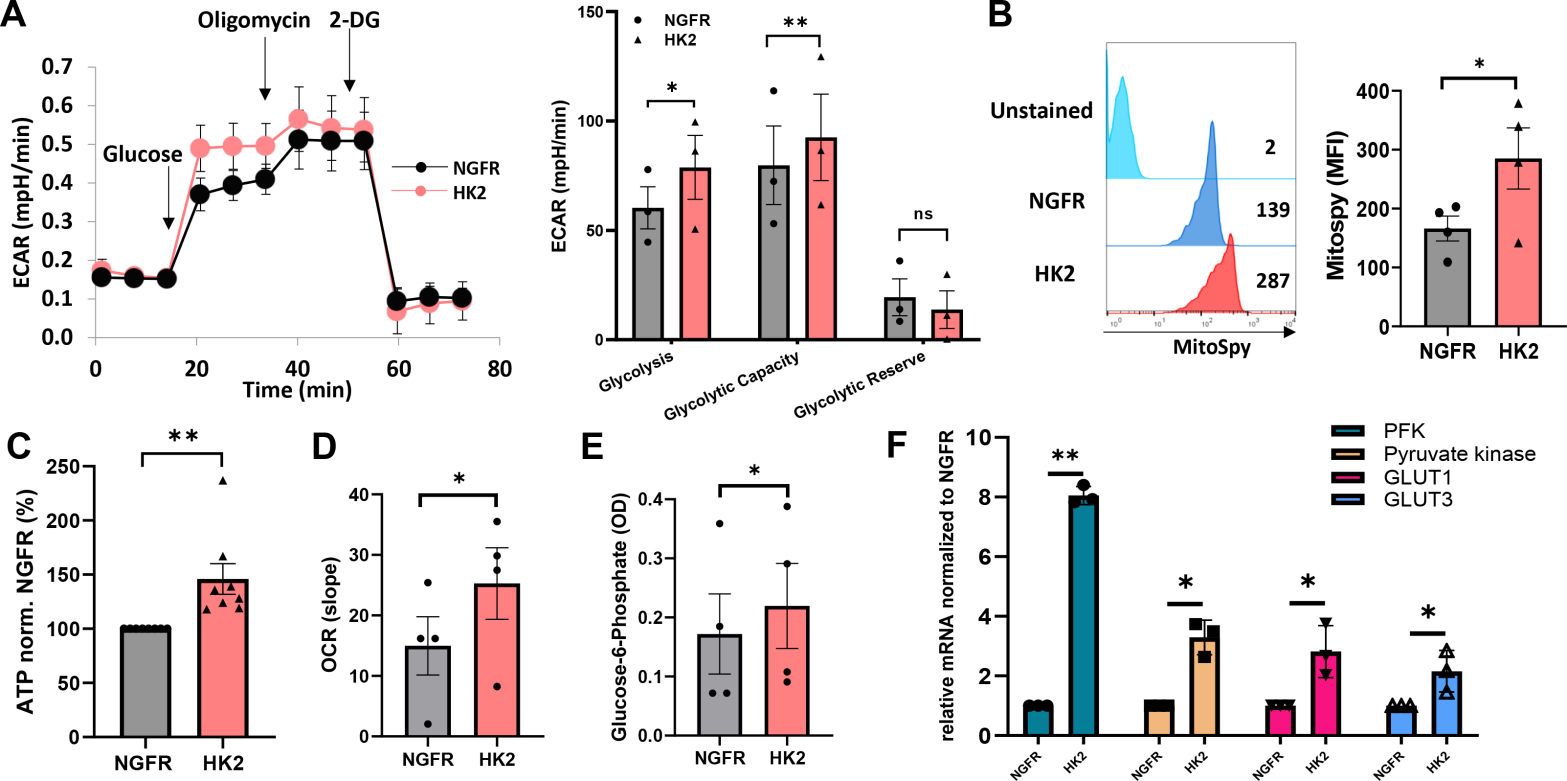
HK2 overexpression can improve glycolysis and metabolic fitness. **(A)** Evaluation of T cell metabolic function using the Seahorse extracellular flux analyzer. Extracellular acidification rate (indicated as ECAR) was measured. The left panel shows a representative experiment and the right panel depicts the mean+SEM of n=3 (**: p<0.01, *: p<0.05 and ns – not significant). **(B)** Mitochondrial mass was measured by FC, gated on NGFR^+^ population using MitoSpy green. The left panel is a representative a result and the right panel is the mean + SEM of n=4 independent experiments with 4 different donors. The difference between HK2 and NGFR (control) was found statistically significant as indicated (*p<0.05, calculated using a *Student’s* paired t-test). **(C)** ATP was measured using the CellTiter-Glo® 2.0 Cell Viability Assay reagent that was added to 1x10^5^ T cells in a 96-well plate. The ATP expression levels were normalized to NGFR. These results are representative of n=8. **(D)** Extracellular Oxygen Consumption Assay was measured kinetically for 2 hours by time-resolved fluorescence (TR-F). The mean+SEM of the slope is shown. **(E)** Glucose-6-Phosphate was measured by colorimetry at 450 nm. The mean + SEM of the optical density (OD) is shown. **(A–E)** These results are performed with at least 3 different donors and the difference between HK2 and NGFR (control) was found statistically significant as indicated (*p<0.05, calculated using a *Student’s* paired t-test). **(F)** RNA from HK2 or NGFR (control) transduced T cells was extracted and reverse-transcribed. PFK, Pyruvate kinase, GLUT1 and GLUT3 transcript expression was measured by qPCR and normalized to control. These results are presented as mean+SEM of n=3.

To further understand the impact of HK2 expression on engineered T cells, we measured the extracellular acidification rate (ECAR) in the culture medium as a surrogate for glycolysis. HK2 overexpression led to a significant increase in glycolysis with an increase of 30% in the HK2 group and in glycolytic capacity (with an increase of 16% in the HK2 group) compared to NGFR (**Figure 2A**). As aforementioned, HK2 plays a crucial role in glucose metabolism and glycolysis. In mitochondria, HK2 interacts with the voltage-dependent anion channel (VDAC), also known as mitochondrial porin, which is located on the outer mitochondrial membrane (24). This interaction facilitates the coupling of glycolysis with mitochondrial ATP generation, as it allows the direct access of ATP produced by glycolysis to the mitochondrial respiratory chain for further energy production (16, 25, 26). We decided to measure the mitochondrial mass of engineered T cells and observed an increase in MFI in the HK2 group compared to the control group (285 vs. 186; **Figure 2B**). Furthermore, we measured the ATP production induced by HK2 forced expression in T cells and observed an increase of 41% compared to control (**Figure 2C**, p<0.01). Oxygen consumption rate (OCR) in HK2-enginereed T cells was also elevated with an increase of 68% compared to control (**Figure 2D**). Finally, we observed a higher level of Glucose-6-phosphate in HK2-transduced T cells (increase of 28% - **Figure 2E**). Given that an increase in glycolytic flux can influence other components of the glycolytic pathway, we assessed the expression of key glycolytic genes, including phosphofructokinase (PFK), pyruvate kinase, GLUT1, and GLUT3. Transcriptional analysis revealed a statistically significant upregulation of these genes in HK2-overexpressing T cells compared to control cells (**Figure 2F**). Specifically, we observed a 2- to 4-fold increase in GLUT1, GLUT3 and pyruvate kinase transcripts. Notably, PFK transcript levels increased by 8-fold, which is particularly significant as PFK is a rate-limiting enzyme in glycolysis; it catalyzes the conversion of fructose 6-phosphate to fructose 1,6-bisphosphate and plays a central role in glycolytic regulation through allosteric inhibition or activation. These findings suggest that HK2 not only enhances glycolysis but also modulates broader aspects of the glycolytic machinery in engineered T cells which may further impact on T-cell functionality as we recently showed (27). Overall, HK2-engineered T-cells exhibited an increased glycolytic capacity and mitochondrial mass which may facilitate energy production and metabolic fitness.

### 3.3 HK2 forced expression enhances cytokine secretion and upregulate activation markers in engineered-T cells

**Figure 3:**
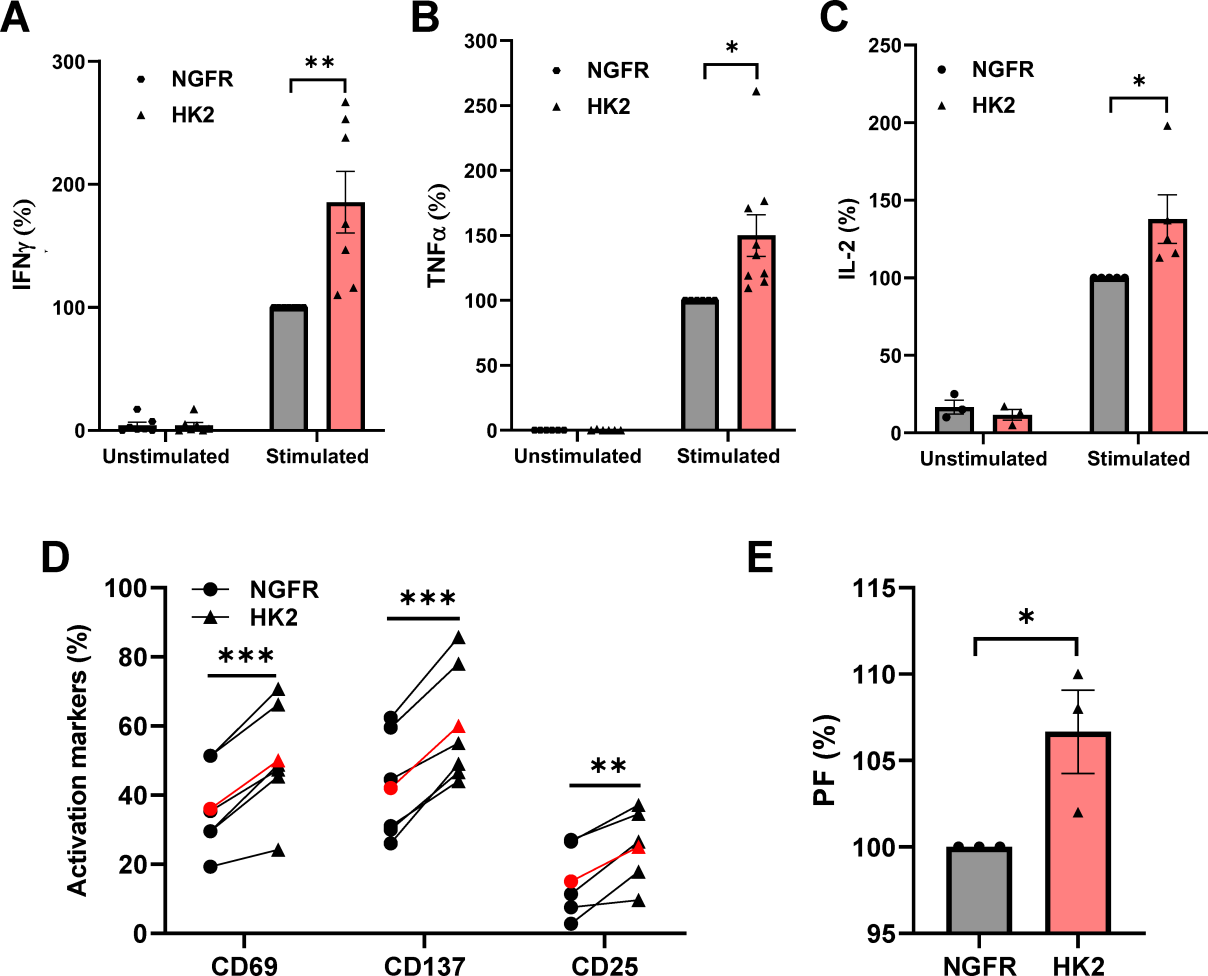
HK2 forced expression enhances cytokine secretion and upregulates activation markers in engineered-T cells. **(A–C)** Primary human T cells were transduced with either NGFR vector (control) or HK2 and were stimulated with plate-bound OKT3. IFNγ **(A)**, TNFα **(B)** and IL-2 **(C)** were secreted in the culture supernatant and measured by ELISA. These results represent the mean+SEM and normalized to control NGFR (with an average secretion of 3.1 ng/mL, 2.6 ng/mL and 0.95 ng/mL for IFNγ, TNFα and IL-2 respectively). **(D)** CD69, CD137, and CD25 expression was measured by flow cytometry, gated on the CD8^+^ population. The mean of n>5 is represented by a red line. **(E)** T cells were labeled with CFSE and stimulated with an anti-CD3 antibody for 4 days. The PF (proliferation factor = MFI day 10/MFI day 14 normalized to NGFR (control) is shown. These results were obtained in n=3 experiments. **(A–E)** These results are performed with at least 3 different donors and the difference between HK2 and NGFR (control) was found statistically significant as indicated (*p<0.05, calculated using a *Student’s* paired t-test). ∗: p < 0.05, ∗∗: p<0.01, ∗∗∗: p < 0.001.

To study the impact of HK2 on T-cell function, we stimulated HK2-engineered human T cells with plate-bound OKT3 antibody. HK2-modified cells exhibited markedly elevated levels of IFNγ, TNFα and IL-2 secretion in comparison to the control cells transduced with NGFR (**Figures 3A–C**). Upon normalizing IFNγ secretion to that observed in the control group (set at 100%, averaging 2.132 ng/mL post-stimulation), we observed an average increase of 72% (p<0.01) in the HK2 group (**Figure 3A**). Likewise, T cells expressing HK2 showed enhanced secretion of TNFα and IL-2 compared to the control group (e.g., 35% and 38% more in the HK2 group for TNFα and IL-2, respectively; **Figures 3B, C**, p<0.05). The increase in these cytokines is particularly significant, as they are known to play critical roles in the anti-tumor activity of T cells (28).

The increase in the expression of activation markers such as CD69, 41BB (CD137) and CD25 is a significant factor in enhancing T-cell function. We evaluated their surface expression on T cells transduced with HK2 or NGFR (control) after OKT3 stimulation. In comparison to the control T cell population, HK2-engineered T cells displayed statistically significant enhanced expression of these markers (**Figure 3D**). We observed an average of 50%, 60% and 25% in CD69, CD137 and CD25, respectively, in the HK2 group compared to 36%, 42% and 15% in the NGFR group (**Figure 3D** p<0.01). Finally, we followed cell proliferation for 4 days. A slight increase was observed in the proliferative capacity (with a proliferation factor based on an MFI ratio of 107 in the HK2 group compared to 100 in the NGFR group, p<0.05; **Figure 3E**).

Thus, HK2-expressing T cells demonstrated improved cytokine secretion capability and activation marker upregulation.

### 3.4 HK2 can improve the anti-tumor function of TCR T cells

**Figure 4:**
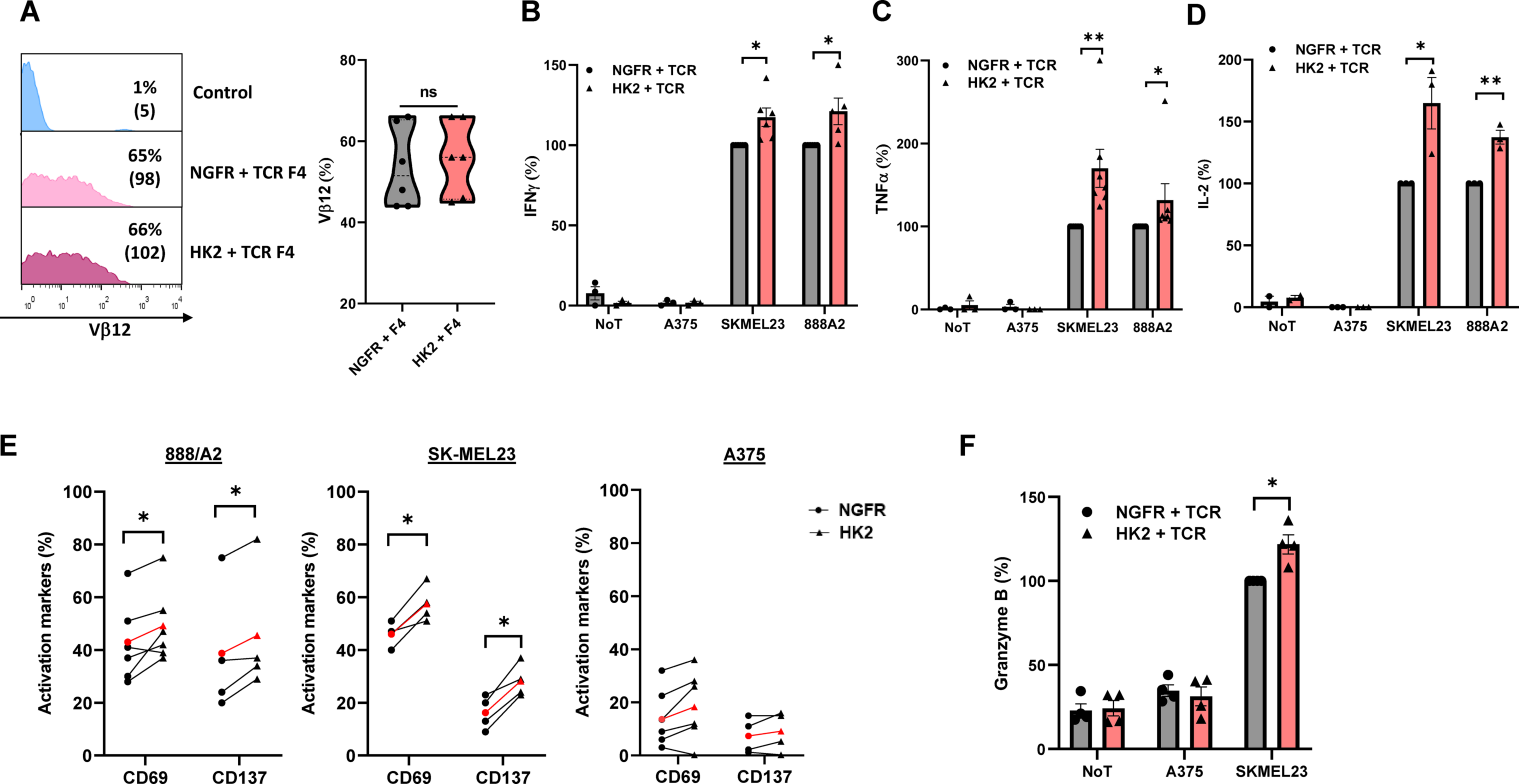
HK2 can improve the anti-tumor function of TCR T cells. **(A)** Human PBLs were transduced with a MART1-specific TCR. After 24 h, the cultures were split and transduced with either HK2 or NGFR. 72 h after transduction, TCR F4 expression was measured by FC using a specific antibody (Vβ12). The left panel is a representative result, and the right panel presents the mean+SEM of n=6. **(B–D)** Following overnight co-culture with melanoma cell lines, IFNγ **(B)**, TNFα **(C)** and IL-2 **(D)** secreted in the culture supernatant were measured by ELISA. These results are the mean+SEM of n>3 and normalized to control (NGFR+TCR with reference concentrations ranging between 5.9-11 ng/ml for IFNγ, 6.6-17.4 ng/ml for TNFα, and 0.23-0.5 ng/ml for IL2). **(E)** CD69 and CD137 expression levels were measured by FC, gated on the CD8^+^ population following overnight co-culture with melanoma cell lines. These results were obtained in n>3 independent experiments. **(F)** Following an overnight co-culture with SKMEL23 cell line, granzyme B secreted in the culture supernatant were measured by ELISA. These results were obtained in n=4 **(A–F)** These results are performed with at least 3 different donors and the difference between HK2 and NGFR (control) was found statistically significant as indicated (*p<0.05, calculated using a *Student’s* paired t-test). ∗∗: p<0.01, ns: not significant.

T cell specificity can be redirected against cancer by engineering them to express exogenous T cell receptors (TCRs) (7). To further study the impact of HK2 expression in an antigen-specific setting, we co-express the HK2 gene along with a MART1-specific TCR termed F4. We expressed TCR F4 concomitantly with HK2 in human T cells and observed a similar TCR expression in all tested groups (65-66%; p>0.05) (**Figure 4A**). Then, these cells were co-cultured with various human melanoma cell lines and measured IFNγ, TNFα and IL-2 secretion (**Figures 4B–D**). We observed a 1.2-1.7-fold increase in cytokine secretion by the HK2 group in co-cultures with 888A2. With SKMEL23 cell line, we observed 70% more TNFα, 65% more IL2 and 17% more IFNγ. No significant cytokine secretion was detected in co-cultures with control A375 or in the absence of targets. Overall, HK2 enforced expression in T cells mediated an enhanced anti-tumor cytokine secretion capability.

Following this, we conducted additional co-cultures with melanoma target cells to evaluate activation markers and granzyme B expression (**Figures 4E, F**). We noted a significant elevation in CD69 and CD137 expression levels in co-culture with melanoma targets. For example, in co-cultures with SKMEL23 and compared to T-cells expressing NGFR (control), a robust increase in CD69 and CD137 expression by HK2 cells was observed (25% and 74%, respectively). Cytotoxic T cells produce and store granzymes in cytotoxic granules, and upon activation by antigen recognition, they release these upon contact with target cells, thereby contributing to inducing apoptosis in infected or cancerous cells (29, 30). We decided to measure their secretion following overnight co-culture and observed an increase of 22% Granzyme B expression in the HK2 group (**Figure 4F**). Thus, HK2-expressing T cells demonstrated improved activation marker upregulation and granzyme expression.

### 3.5 HK2 improves T cell anti-tumor function *in vitro* and *in vivo*

**Figure 5:**
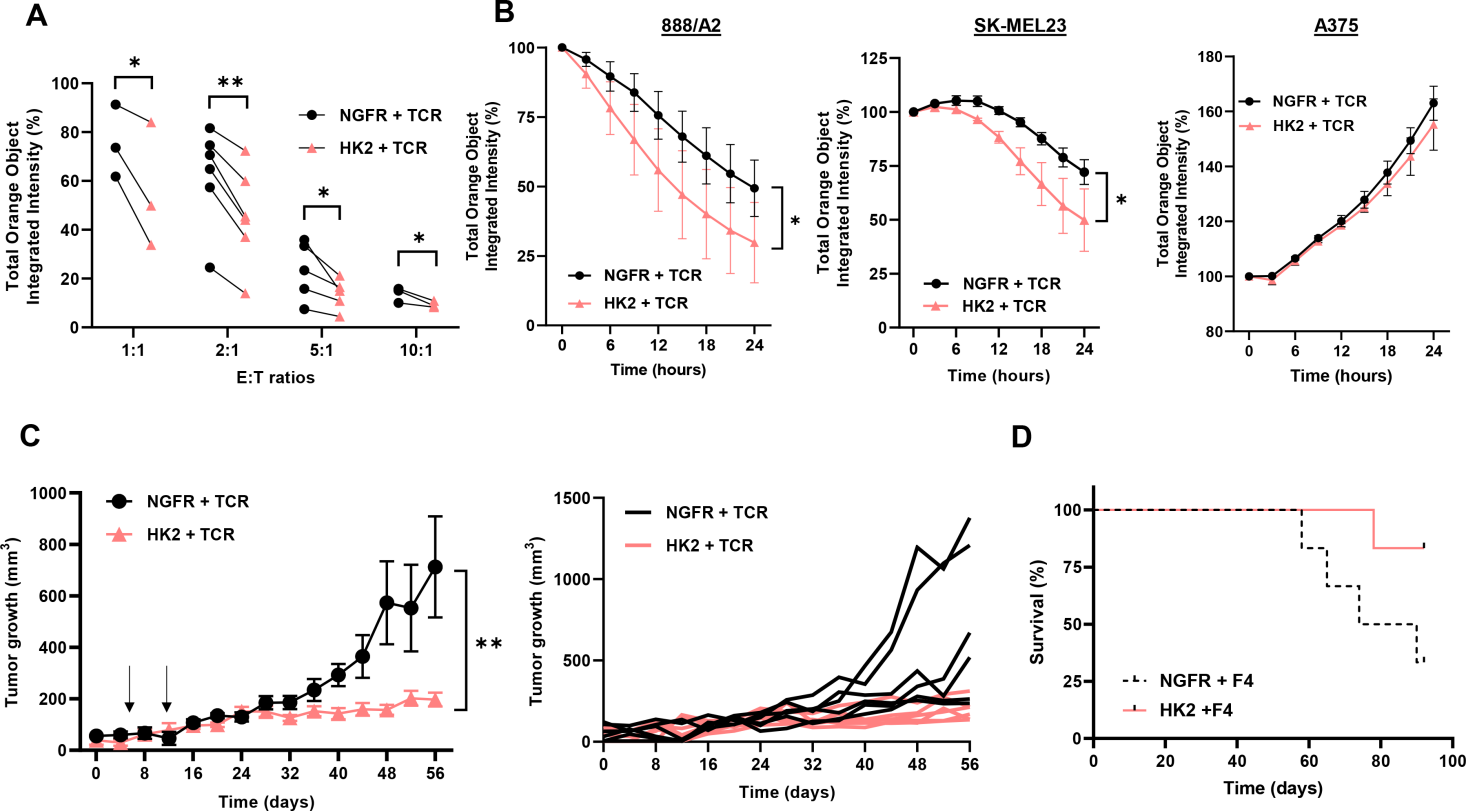
HK2 improves T cell anti-tumor function *in vitro* and *in vivo.* **(A)** NGFR + TCR and HK2 + TCR engineered T-cells were co-cultured with tumor cells (888A2) at different E:T (Effector : Target) ratio and incubated in an Incucyte apparatus. mCherry^+^ live cell population was followed over time and normalized to time=0. These results are the mean+SEM of n>3 independent experiments performed with at least 3 different donors. The difference between NGFR vector (control) and HK2 was found statistically significant as indicated (*p<0.05 and **p<0.01 calculated using a *Student’s* paired t-test). **(B)** Representative experiments are shown in co-culture, with 888A2, SKMEL23 and A375 (negative control) melanoma cell lines at 1:1 E:T ratio. **(C)** NSG mice were inoculated with 2x10^6^ 888A2 tumor cells. Then, 5x10^6^ of NGFR + TCR or HK2 + TCR-engineered T cells were IV injected in mice, one week and two weeks after tumor establishment (indicated by arrows). Tumor growth was measured in a blinded fashion using a caliper. Curves for the tumor growth average (n=6) and spider plots of the tumor volumes are shown. The difference between NGFR vector (control) and HK2 was found statistically significant as indicated (calculated using a *Student’s* paired t-test). **(D)** The survival of the respective groups is represented by a Kaplan-Meier curve (p=0.07, calculated using a *Log-Rank* test).

To further examine the function of HK2-engineered T cells, we conducted a cell-mediated cytotoxicity assay, evaluating live melanoma target cells following a 24-hour co-culture with T cells at various Effector: Target (E:T) cell ratios (**Figure 5A**). Superior cytotoxicity for HK2-transduced cells was evident across all ratios, with the most pronounced effects observed at 1:1 and 1:2 ratios. A notable reduction was observed in the number of live 888A2 cells after 24 hours, with only 30% viability in the HK2 group compared to 49% in the NGFR group. Similar results were obtained in co-cultures with SKMEL23, with a significant reduction of live target tumors (from 72% to 50%) between the control and the HK2 group respectively (**Figure 5B**). No significant cytotoxicity activity was observed against the A375 cell line.

Finally, we assessed the *in vivo* anti-tumor function of HK2-transduced T cells and examined the ability of the latter to influence tumor growth in a human tumor xenograft mouse model. For this purpose, 2x10^6^ tumor cells (888A2) were injected into the flank of NSG mice. 5x10^6^ T cells (HK2 + TCR or NGFR + TCR) were injected through the tail vein, one and two weeks after the tumor cell injection. We followed tumor growth and noted that HK2 + TCR-T cells mediated a significant delay in tumor growth compared to the control group that was treated with NGFR + TCR-transduced T cells (**Figure 5C**; n=6, p<0.01). Moreover, at the experiment endpoint, 83% of the HK2-treated mice survived compared to 33% in the control group (**Figure 5D**). In conclusion, HK2 engineered T cells could delay tumor growth and significantly prolong the survival of tumor-bearing mice.

## 4 Discussion

In this study, we have demonstrated the feasibility of genetically engineering T cells to overexpress HK2, a key and the first enzyme in the glycolysis pathway, to enhance their anti-tumor efficacy. Previous research has emphasized the critical role of glucose availability in the tumor microenvironment (TME) for optimal T cell function (31). Low glucose levels within the TME have been associated with impaired T cell proliferation, cytokine production, and cytotoxicity, hampering effective immune responses against tumors (32). While existing studies have primarily focused on the limited glucose accessibility to T cells within the TME, recent evidence suggests that tumor-infiltrating T cells may exhibit defective glycolysis despite accessing similar glucose levels as tumor cells (33). Still, we and others have recently demonstrated that enforcing glucose transporter expression improves T-cell metabolic fitness and anti-tumor activity (27, 34–37). Additionally, functional enhancement and increased glycolytic rates could be achieved in T-cells by culturing them in high glucose conditions (38). Our study complements these findings by targeting HK2, a critical enzyme upstream in glycolysis, to directly modulate metabolic flux and improve T-cell functionality. While glucose transporters primarily enhance glucose uptake, HK2 engineering provides a broader metabolic advantage by increasing glycolytic and mitochondrial fitness, offering a complementary approach to improving T-cell efficacy in cancer immunotherapy. Indeed, we recently showed the feasibility of co-expressing in tumor-specific T-cells a bottleneck glycolytic enzyme, namely phosphofructokinase (PFK) together with GLUT3 and this led to superior anti-tumor activity (27).

Our investigation targeted HK2, recognizing its pivotal role in driving glycolysis and cellular metabolism (16). By enhancing HK2 expression in T cells, we aimed to maintain their glycolytic capacity and consequently improve their immune fitness within the TME. We observed increased HK2 mRNA, HK2 protein expression, as well as glucose-6-phosphate levels and enhanced glycolytic flux, though these may not exclusively reflect HK2 activity. Future studies may include enzymatic assays to confirm HK2 activity and its direct contribution to glycolysis in engineered T cells. HK2 activity enhances glycolysis, enabling T cells to utilize glucose for generating biosynthetic precursors such as amino acids and nucleic acids, which are essential for cytokine production, effector molecule synthesis, rapid growth, and population expansion through the pentose phosphate pathway (PPP) (39). Notably, NADPH production via the PPP plays a crucial role in the synthesis of amino acids, nucleic acids, and fatty acids, and the higher glycolytic flux supported by HK2 likely provides a faster throughput for this pathway compared to mitochondrial respiration (39). Additionally, the increased glycolytic flux driven by HK2 may activate mTOR signaling, thereby promoting the expression of activation markers (e.g., CD25, CD69) and facilitating differentiation into potent cytotoxic effector cells (40).

HK2 function in T-cells has been shown to be critical in autoimmune pathologies such as rheumatoid arthritis (41); this strengthened our assumption that it also can bear an important role in generating a potent anti-tumor response as the latter could also be considered to some extent of autoimmune nature. Moreover, beyond its role in glycolysis, HK2 has been implicated in regulating mitochondrial function, which plays a critical role in cellular metabolism and energy production (42). Additionally, mitochondrial HK2 impacts glycolysis and ROS regulation, contributing to Ca2^+^ signaling/homeostasis for fine-tuning cellular bioenergetics, survival, and triggering autophagy under nutrient-deprived conditions (16). Therefore, future studies may investigate the role of HK2 in modulating mitochondrial function in T cells and its implications for anti-tumor immunity. Interestingly, previous reports have shown in the past that conditional deletion of HK2 in T cells did not significantly affect their development, survival, or function in certain *in vitro* and *in vivo* models (43, 44). This discrepancy may be attributed to fundamental differences in experimental design, biological context, and the T-cell subsets examined. Both aforementioned studies utilized HK2 knockout models and primarily focused on CD4^+^ T-cell function in non-tumor settings, such as viral infection and inflammation. They demonstrated that compensatory upregulation of HK1 could maintain glycolysis and sustain CD4+ T-cell function under these conditions. However, CD8^+^ T cells, which are more reliant on glycolytic metabolism to fuel their cytotoxic activity, may depend more critically on HK2 in the tumor microenvironment. In contrast, by employing HK2 overexpression, we demonstrated that increased glycolytic flux significantly enhanced T-cell cytotoxicity, particularly in the nutrient-restricted and hypoxic conditions of the tumor microenvironment. Altogether, these findings may show the dispensability of HK2 in maintaining baseline immune responses but reveal its critical role in amplifying anti-tumor activity in environments with heightened metabolic demands. Nonetheless, while this study focused on a distinct goal, further exploration into the context-dependent role of HK2, its interplay with other metabolic regulators, and its broader implications for T-cell responses in the tumor microenvironment will be essential to fully understand its impact on T-cell function.

Our study primarily focused on a well-established TCR/melanoma model, but future research should explore the applicability of HK2-engineered T cells in other solid tumors known for their high glycolytic activity such as pancreatic or breast tumors (45). Recent head-to-head comparison of CD19 CAR T-cells activity suggests that CD28-based CARs mediated better clinical outcome than 41BB-based receptors, though with more common side effects (46). Both CAR CD28 and 41BB signaling pathways may enhance glycolysis. Still, CAR CD28 signaling has been shown to promote a more efficient and sustainable glycolytic metabolism. It was demonstrated that CAR CD28-engineered T cells exhibited enhanced glycolytic capacity and metabolic fitness compared to CAR 41BB-engineered T cells, resulting in superior anti-tumor efficacy (47). Moving forward, it will also be crucial to assess the long-term effects of HK2 overexpression in T cells. Whereas the xenograft model we used allows specific testing of human tumor-specific engineered T cells, it has limitations, including the absence of an immune-competent microenvironment. Alternative models, such as syngeneic or humanized mouse models, could complement this approach in future studies to assess broader immune interactions. Addressing these questions will be important for the safe and effective translation of HK2-engineered T-cell therapy into clinical practice.

T-cell metabolism can be manipulated to enhance their effectiveness and therapeutic potential. This can be achieved by delivering metabolic regulators directly into the tumor microenvironment or preconditioning T cells in optimized media or growth conditions prior to transfer (48). However, the long-term efficacy of these strategies remains uncertain, as T cells may revert to their original metabolic state post-transfer. In this study, we genetically engineered T cells to address several of these challenges, facilitating the development of consistently potent effector T cells. Additional genetic engineering strategies, such as CRISPRa, could further expand these advancements (49). We hypothesize that this metabolic enhancement approach could be applied not only to T cells engineered with tumor-specific receptors but also to naturally occurring tumor-infiltrating lymphocytes (TILs) or other immune cells, such as B cells or natural killer (NK) cells, for therapeutic applications. While challenges remain, including scaling up the manufacturing of HK2-engineered T cells for clinical applications and accounting for patient-to-patient variability, future studies should evaluate their therapeutic potential in clinical trials across various solid tumors.

HK2-engineered T cells have the potential to significantly improve patient outcomes by enhancing tumor control and prolonging survival. These cells could be particularly beneficial in aggressive solid tumors where metabolic competition severely impairs immune responses, such as in brain or pancreatic cancers. Combining HK2 overexpression with immune checkpoint inhibitors or metabolic modulators could yield synergistic effects. Furthermore, comprehensive mapping and omics analyses of different tumor types could help identify metabolic pathways that can be targeted in T cells to further enhance their anti-tumor function (50).

While this study focuses on the role of HK2 in modulating T-cell metabolism and function, it is important to acknowledge that HK2 also plays a pivotal role in regulating the metabolic environment of tumor cells. HK2 is overexpressed in many cancers and is a key driver of glucose metabolic reprogramming, which supports tumor proliferation, survival, and immune evasion. For example, HK2-mediated glycolysis contributes to the production of metabolic intermediates required for biosynthesis and energy generation in tumor cells, while simultaneously impairing immune cell function by depleting glucose availability in the tumor microenvironment (51, 52). Additionally, HK2 has been implicated in promoting immune evasion by enhancing PD-L1 expression on tumor cells, further dampening anti-tumor immune responses (53). This dual role of HK2 highlights its significance as a therapeutic target, with potential implications for cancer metabolism and immunotherapy.

In conclusion, our study shows that enhancing the expression of the HK2 enzyme enhances T cell metabolic fitness, improving their function *in vitro* and in an animal model. We believe that such a strategy, focused on manipulating immunometabolism, holds significant promise for improving cellular immunotherapy.

## Data Availability

The original contributions presented in the study are included in the article/supplementary material. Further inquiries can be directed to the corresponding author.
